# Combined anomalous origin of a left inferior thyroid artery and a left vertebral artery: a case report

**DOI:** 10.1186/1757-1626-2-7400

**Published:** 2009-05-26

**Authors:** Konstantinos Natsis, Matthaios Didagelos, Georgios Noussios, Aspasia Adamopoulou, Elisavet Nikolaidou, Georgios Paraskevas

**Affiliations:** Department of Anatomy Medical School of Aristotle University of ThessalonikiP.O. Box: 300, P. Code: 54124, ThessalonikiGreece

## Abstract

An abnormal origin of a left inferior thyroid artery from the left vertebral artery that in turn originated from the aortic arch was observed on a 72-year-old Caucasian male cadaver during a dissection anatomy practice. We describe in detail the morphology of this extremely rare anatomical variation and refer to its clinical importance.

## Introduction

Variations have been described for almost every artery of the human body each one having more or less clinical significance, except from the pure anatomical interest [1].

The inferior thyroid artery usually originates from the thyrocervical trunk. However it may also originate from the subclavian artery and rarely from the common carotid artery, aortic arch, brachiocephalic trunk, internal thoracic artery, pericardiophrenic artery, vertebral artery or as a common stem with the contralateral inferior thyroid, dorsal scapular and suprascapular arteries [1].

The left vertebral artery usually originates from the first part of the subclavian artery but it may also arise from the left common carotid artery, from the root of the subclavian, close to the aortic arch, or directly from the aortic arch [1].

## Case presentation

During an anatomy practice, at the Department of Anatomy of the Medical School of Aristotle University of Thessaloniki, a 72-year-old, Greek, Caucasian, formaline-embalmed male cadaver was dissected, in accordance with all legal requirements posed by the University's Ethical Committee, and a rare anatomical variation in the vessels of the aortic arch was observed. His height was 1.77 m and his weight 85 kg. There were no pathological conditions concerning the neck and the mediastinum vessels. The cause of death was myocardial infarction. The complete medical history of the cadaver was not available.

**Figure 1.**
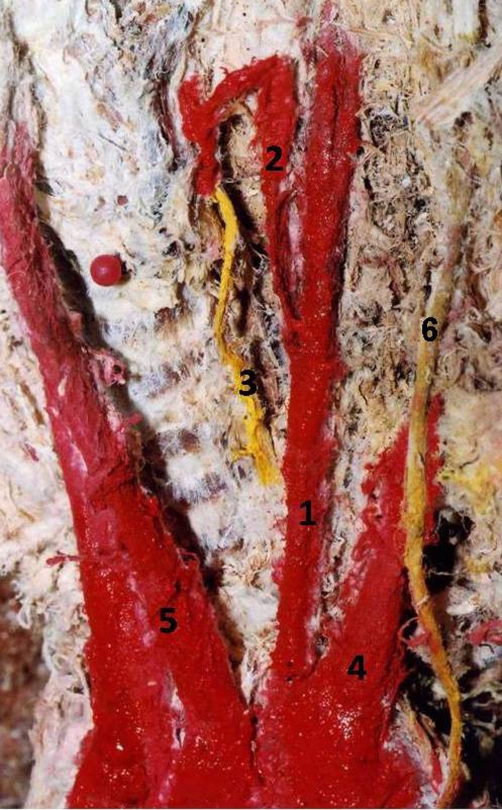
Cadaveric photo after highlighting in colour the anatomical structures of interest. Left vertebral artery **(1)** arising from the root of the left subclavian artery **(4)**, close to the aortic arch and behind the left common carotid artery **(5)**. The left inferior thyroid artery **(2)** originates from the left vertebral and supplies the thyroid gland. The recurrent laryngeal nerve **(3)** originating from the vagus nerve **(6)** can also be seen emerging behind the inferior thyroid artery.

**Figure 2.**
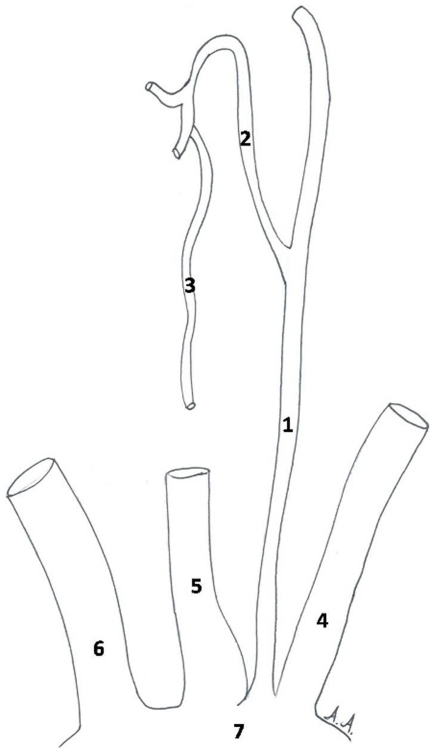
Schematic drawing explaining the anatomical structures described on Figure 1. **(1)** Left vertebral artery, **(2)** Left inferior thyroid artery, **(3)** Recurrent laryngeal nerve, **(4)** Left subclavian artery, **(5)** Left common carotid artery retracted to the right side, **(6)** Brachiocephalic trunk, **(7)** Aortic arch.

After resection of the anterior thoracic wall and anatomical dissection of the ascending aorta and the great vessels arising from it, we observed that the left vertebral artery originated from the root of the left subclavian artery, close to the aortic arch. Following its route upwards to the neck we came across another arterial branch that arose from it having an upward and to the midline course. Carefully dissecting this smaller artery from the surrounding connective tissue we reached the thyroid gland, concluding that it was the inferior thyroid artery. The recurrent laryngeal nerve was found deep to it and it was also revealed. The observations were photographed and documented on diagrams (Figures 1 and 2).

In conclusion the morphology of the aortic branches (from right to left) was:

1. Brachiocephalic trunk, with right subclavian and right common carotid artery.
2. Left common carotid artery.
3. Left vertebral artery, in touch with the root of the left subclavian artery and giving rise higher to the left inferior thyroid artery.
4. Left subclavian artery.

The remaining vasculature of the upper thorax and the neck was according to the normal pattern.

## Discussion

The variations reported on the aortic arch branching are numerous. There may be from one to six branches. What is commonly described as “normal” aortic arch is that giving rise to three branches: 1. brachiocephalic trunk, which in turn branches to the right subclavian and to the right common carotid artery, 2. left common carotid artery and 3. left subclavian artery [1,2].

The vertebral artery arises from the superoposterior aspect of the first part of the subclavian artery. It passes through the foramina in the transverse processes of all of the cervical vertebrae except from the seventh one, curves medially behind the lateral mass of the atlas and enters the cranium via the foramen magnum. Occasionally it may enter the cervical vertebral column via the fourth, fifth or seventh cervical vertebra [2]. The left vertebral artery arising from the aortic arch is the third most common aortic arch branching pattern with an incidence of 0.79-8% [1,3,4]. This variation plays an important role for vascular surgeons, neurosurgeons and thorax surgeons because vertebral artery injury is a known complication of the extended lateral decompression during anterior cervical spine surgery, which can result in exsanguinations and permanent neurologic deficits [5]. Moreover, the vertebral artery may be wrongly considered to be occluded or diseased, either by eluding catheterization during angiography or by lying outside the region of interest during noninvasive studies such as CT angiography, MR angiography or Doppler sonography [6,7]. There is also a report concluding that left vertebral artery of aortic origin is associated with a predilection for vertebral artery dissection in comparison to vertebral artery of subclavian artery origin [8].

The inferior thyroid artery normally arises from the thyrocervical trunk, a branch of the subclavian artery. It loops upwards anterior to the medial border of the scalenus anterior, turns medially just below the sixth cervical transverse process, then descends on longus colli muscle to the lower border of the thyroid gland. It passes anterior to the vertebral vessels and posterior to the carotid sheath and its contents. On the left side, near its origin, the artery is crossed anteriorly by the thoracic duct as the latter curves inferolaterally to its termination [2]. The inferior thyroid artery has a variable branching pattern and is closely associated with the recurrent laryngeal nerve. The nerve can be found deep to the inferior thyroid artery, superficially or between branches of the artery [9,10]. Furthermore, at the level of the inferior thyroid artery, branches of the recurrent laryngeal nerve that are extralaryngeal may be present. Preservation of all those branches is important during thyroidectomy [11].

According to the literature the inferior thyroid artery may originate from the vertebral artery in 0.7% [12] of individuals. No certain clinical impact of this variation is described. However it should be known by neck surgeons in order to avoid implications during thyroidectomy, while trying to ligate the regional vessels. Vascular interventionalists and angiographers should also bear in mind this variation during inferior thyroid artery catheterization either diagnostic or therapeutic, in case of an aneurysm or a rupture at the thyroid region [13,14].

Although many variations of the vertebral and the inferior thyroid artery alone have been reported a combined variation of these arteries is extremely rare. The case described here, with the left inferior thyroid artery arising from the left vertebral artery originating in turn from the aortic arch, is mentioned only by Adachi in his classical cadaveric studies and by Sartor in an angiographic case report [12,15].

## List of abbreviations

CT: Computer Tomography
MR: Magnetic resonance
